# Examining Humans’ Problem-Solving Styles in Technology-Rich Environments Using Log File Data

**DOI:** 10.3390/jintelligence10030038

**Published:** 2022-06-30

**Authors:** Yizhu Gao, Xiaoming Zhai, Okan Bulut, Ying Cui, Xiaojian Sun

**Affiliations:** 1 Department of Educational Psychology, University of Alberta, Edmonton, AB T6G 2G5, Canada; bulut@ualberta.ca (O.B.); yc@ualberta.ca (Y.C.); 2 Department of Mathematics, Science, and Social Studies Education, University of Georgia, Athens, GA 30602, USA; Xiaoming.Zhai@uga.edu; 3 School of Mathematics and Statistics, Southwest University, Chongqing 400715, China; sunxiaojian@swu.edu.cn

**Keywords:** problem-solving style technology-rich environments, experiential learning theory, *k*-means clustering, explanatory item response modeling, log file data

## Abstract

This study investigated how one’s problem-solving style impacts his/her problem-solving performance in technology-rich environments. Drawing upon experiential learning theory, we extracted two behavioral indicators (i.e., planning duration for problem solving and human–computer interaction frequency) to model problem-solving styles in technology-rich environments. We employed an existing data set in which 7516 participants responded to 14 technology-based tasks of the Programme for the International Assessment of Adult Competencies (PIAAC) 2012. Clustering analyses revealed three problem-solving styles: *Acting* indicates a preference for active explorations; *Reflecting* represents a tendency to observe; and *Shirking* shows an inclination toward scarce tryouts and few observations. Explanatory item response modeling analyses disclosed that individuals with the *Acting* style outperformed those with the *Reflecting* or the *Shirking* style, and this superiority persisted across tasks with different difficulties.

## 1. Introduction

As information and communication technologies rapidly integrate into people’s everyday lives, the importance of being able to use technological tools to solve problems continues to grow in recent years (Hämäläinen et al. 2015; Koehler et al. 2017; Zheng et al. 2017). As highlighted by Iñiguez-Berrozpe and Boeren (2020), being insufficient to solve technology-based problems can exclude one from the labor market. This has been particularly true when people felt challenged to use computers or other digital devices to perform work-related activities (Hämäläinen et al. 2015; Ibieta et al. 2019; Nygren et al. 2019; Tatnall 2014). Nonetheless, a huge amount of people seem to have insufficient problem-solving performance in technology-rich environments (TRE). As pointed out by Nygren et al. (2019), more than 50% of European aged 16–64 years old were deficient in coping with practical tasks in TRE (e.g., communicating with others by email). Notably, TRE incorporate diverse, versatile, and constantly evolving digital technologies, leading to difficulties in being operated expertly. Considering feasibility reasons, TRE in the present study are limited to settings involving the most common digital technologies (Nygren et al. 2019): computers (e.g., spreadsheet) and Internet-based services (e.g., web browser). To boost the use of digital technologies, a bulk of research has investigated factors that might affect humans’ problem-solving performance in TRE (e.g., Liao et al. 2019; Millar et al. 2020; Nygren et al. 2019; Ulitzsch et al. 2021). Among those findings, problem-solving style was regarded as one of the most prominent factors (e.g., Koć-Januchta et al. 2020; Lewis and Smith 2008; Treffinger et al. 2008).

Problem-solving style describes pervasive aspects of individuals’ natural dispositions toward problem solving. According to Selby et al. (2004, p. 222), problem-solving styles are “consistent individual differences in the ways people prefer to plan and carry out generating and focusing activities, in order to gain clarity, produce ideas, and prepare for action”. This broadly accepted definition indicates that problem-solving style derives from one’s distinguishable behavioral pattern (e.g., He et al. 2021; Ulitzsch et al. 2021). In this regard, problem-solving styles in TRE reflect individuals’ dispositions regarding how they are inclined to interact with surrounding technology environments. Implicit tendencies, in turn, can be partially explicated by behavioral indicators recorded in computer-generated log files, such as timestamps, clicks, and sequence of actions (Bunderson et al. 1989; Eichmann et al. 2019; Oshima and Hoppe 2021). In other words, a critical empirical avenue to profiling an individual’s problem-solving style in TRE is to analyze log file data collected in computer-based problem-solving assessments.

This study analyzed log file data of the Programme for the International Assessment of Adult Competencies (PIAAC) 2012 to unpack problem-solving styles in TRE and examined how problem-solving styles were associated with participants’ performance on TRE-related tasks. In PIAAC 2012, a total of 14 tasks were administered to assess participants’ problem-solving competencies in TRE, all of which simulate real-world problems that adults likely encounter when using computers and Internet-based technologies. The data from assessment tasks provide rich information, such as performance and behavioral information. However, abstracting the useful information from the log files is challenging because multiple variables with manifold types are embedded in the data structure (Han et al. 2019). To overcome this challenge, we first applied clustering techniques to multiple behavioral indicators derived from the 14 tasks, thereby partitioning participants into discrepant clusters. Each cluster was further analyzed and its specific problem-solving style was identified according to behavioral indicators. Finally, we examined how the personal features (i.e., problem-solving style) and their interaction with task features (i.e., task difficulty level) account for participants’ task performance by explanatory item response modeling (EIRM; De Boeck and Wilson 2004).

### 1.1. Problem-Solving Styles in TRE

In this study, the problem-solving style in TRE is conceptualized and operationalized as the consistent individual behavior in planning and carrying out problem-solving activities in surrounding technology environments (Isaksen et al. 2016; Selby et al. 2004; Treffinger et al. 2008). Despite the importance and the pervasiveness of problem-solving styles, few pertinent theories have been put forward in this area. A potential theory that may enlighten our understanding of problem-solving styles in TRE is experiential learning theory (Kolb 2015). Experiential learning theory emphasizes the central role of experience in human learning and development processes and has been widely accepted as a useful framework for educational innovations (Botelho et al. 2016; Koivisto et al. 2017; Morris 2020). In his seminal works, Kolb (2015) suggests four types of learning modes to portray individuals’ learning preferences as a combination of grasping and transforming experiences: if individuals prefer an abstract grasping of information from experiences, their inclined learning mode is abstract conceptualization (AC); in contrast, if individuals prefer highly contextualized and hands-on experiences, their learning mode is known as concrete experience (CE); if individuals prefer to act upon the grasped information, their preference of transforming experience is active experimentation (AE); otherwise, their preferred way may be reflective observation (RO). Thereafter, much research has studied learning styles based on individuals’ relative preferences for the four learning modes and agrees upon a nine-style typology (e.g., Eickmann et al. 2004; Kolb and Kolb 2005a; Sharma and Kolb 2010). Specifically, four learning styles emphasize one of the four learning modes; another four represent learning style types that emphasize two learning modes; one learning style type balances all the four learning modes. For example, learning styles of *Acting* and *Reflecting* correspond to learning modes of AE and RO, respectively. Individuals with the *Acting* style usually possess highly developed action skills while utilizing little reflection (AE). In contrast, those with the *Reflecting* style spend much time buried in their thoughts, but have trouble putting plans into action (RO).

Learning modes are highly associated with problem-solving styles. There is an emerging consensus that learning interacts with and contributes to ongoing problem-solving processes (Ifenthaler 2012; Wang and Chiew 2010). Research has indicated that problem solving is not only a knowledge application process but also a knowledge acquisition and accumulation process. In this respect, humans’ learning modes along with exploring problem environments can be part of problem-solving styles (Kim and Hannafin 2011). For example, Romero et al. (1992) developed the Problem-Solving Style Questionnaire based on a hypothesized problem-solving process in which the four learning modes (i.e., CE, RO, AC, and AE) are involved. Besides the close conceptual connections between learning modes and problem-solving styles, learning modes are increasingly incorporated into designing technology-enhanced learning environments given their capability to describe users’ online learning styles. For example, Richmond and Cummings (2005) discussed the integration of learning modes with online distance education and suggested that learning modes should be considered for instructional design to ensure high-quality online courses and to achieve positive student outcomes. In addition, an earlier study by Bontchev et al. (2018) has demonstrated the usefulness of learning modes in enlightening humans’ styles in game-based problem solving. Therefore, learning modes can potentially inform the types of problem-solving styles in TRE.

### 1.2. Acting and Reflecting Styles

Among learning styles portrayed in a two-dimensional learning space defined by AC-CE and AE-RO, the *Acting* and *Reflecting* styles are particularly representative of individual interactive modes in TRE. For example, Hung et al. (2016) took the *Acting* and *Reflecting* styles into account when they provided adaptive suggestions to optimize problem-solving performance in computer-based environments. Bontchev et al. (2018) investigated problem-solving styles within educational computer games, which correspond to the *Acting* and *Reflecting* styles. These studies confirmed that the *Acting* and *Reflecting* styles are feasible to describe problem-solving styles in TRE.

A distinctive feature of the *Acting* style is the strong motivation for goal-directed actions that integrate people and objects (Kolb and Kolb 2005b). Individuals with the *Acting* style prefer to work and try objects out (Hung et al. 2016). Within TRE, individuals with the *Acting* style habitually perform actions quickly and frequently, which implies their intuitive readiness to act. In contrast, the *Reflecting* style is characterized by the tendency to connect experience and ideas through sustained reflections (Kolb and Kolb 2005b). Individuals with the *Reflecting* style prefer to evaluate and think about objects (Hung et al. 2016). When interacting with objects in TRE, they need time to observe and establish the meaning of available operations in technological environments. They watch patiently rather than automatic reaction and wait to act until certain of their intention.

In addition to their suitability for describing problem-solving styles in TRE, evidence shows that the *Acting* and *Reflecting* styles are relevant to problem-solving performance. For example, Kolb and Fry (1975, p. 54) suggested that a behaviorally complex learning environment distinguished by “environmental responses contingent upon self-initiated action” emphasizes actively applying knowledge or skills to practical problems, and thus better supports the learning mode of AE. Following this view, individuals with the *Acting* style are supposed to have better performance in TRE-related tasks than those with the *Reflecting* style who have deficiencies in AE. However, this theoretical assumption needs to be empirically examined.

Furthermore, it is crucial to consider the role of problem characteristics (e.g., problem type or problem difficulty) in the relationship between individuals’ problem-solving styles and their performance in problem solving. As stated by Treffinger et al. (2008), an individual’s preference for a certain problem-solving style can influence his or her behavior in finding, defining, and solving problems. That is, a certain problem-solving style can either hamper or facilitate problem-solving performance, depending on some characteristics of problems. For example, Treffinger et al. (2008) found that individuals with the explorer style deal well with ill-defined and ambiguous problems, while individuals with the developer style are adept at handling well-defined problems. Thus, studies need to examine the role of problem characteristics when investigating the impact of problem-solving styles on problem-solving performance.

### 1.3. Behavioral Indicators of Acting and Reflecting Styles in TRE

To examine the feasibility of the *Acting* and *Reflecting* styles in describing problem-solving behaviors in TRE, two behavioral indicators were abstracted from log files: duration of planning period at the beginning of the problem-solving process and interaction frequency during the entire problem-solving process. For simplicity, the two behavioral indicators were abbreviated as planning duration and interaction frequency, respectively. Planning duration denotes the period from the time that a task starts to the point that people take their first action to perform the task. It is also called first move latency (e.g., Albert and Steinberg 2011; Eichmann et al. 2019) or timing of the first action (e.g., Goldhammer et al. 2016; Liao et al. 2019). In this study, the term “planning duration” is used to emphasize people’s thinking and reflection on the problem at hand (Albert and Steinberg 2011). Interaction frequency indicates how frequently people interact with a task during the period from the first action to the end of the task.

The two indicators formulate a two-dimensional space that could portray individuals’ problem-solving behaviors. Specifically, based on previous research (e.g., Eickmann et al. 2004; Hung et al. 2016; Kolb and Kolb 2005a), individuals with the *Acting* style prefer to act on tasks with multiple trials while seldom reflecting on their behaviors during the course. They perform like experimentalists. In contrast, those with the *Reflecting* style prefer to fully reflect on situations instead of taking concrete actions. They tend to be theoreticians. During problem solving in TRE, individuals with the *Acting* style usually spend less time on planning, but interact more with objects in comparison with those with the *Reflecting* style who spare more time for planning, but execute tasks less.

Although the role of planning duration and interaction frequency in problem solving has been widely studied previously (Albert and Steinberg 2011; Eichmann et al. 2019; Greiff et al. 2016), no study has explored how these two measures together inform individual problem-solving styles in TRE. Albert and Steinberg (2011) found that planning time, which reflects self-regulatory control, strongly and positively predicted outcomes of problem solving. However, a longer time of first-move latency may not necessarily indicate participants as being more thoughtful. Instead, participants may merely feel confused about problems (Zoanetti and Griffin 2014). In fact, interaction frequency could cooperate with planning duration in inferring participants’ inclination toward problem solving in TRE (Eichmann et al. 2019). For example, a thoughtful individual would not only spend more time planning at the beginning but also have relatively fewer tryouts during the problem-solving process, indicating their accurate reasoning and confident judgments.

### 1.4. Current Study

Given the limited volume of research on humans’ problem-solving styles in TRE, this study first examined *Acting* and *Reflecting* styles in TRE using two indicators: planning duration and interaction frequency. We then compared different problem-solving styles to identify the most desirable one for solving technology-based problems. Finally, we examined how task difficulty moderates the relationship between individual task performance and individual problem-solving styles. The study answers three research questions:

1. Did participants demonstrate *Acting* or *Reflecting* problem-solving styles when solving problems in TRE?
2. If so, which problem-solving style better favors participants’ performance?
3. How did task difficulty moderate the relationship between participants’ problem-solving styles and their performance on TRE-related tasks?

## 2. Materials and Methods

### 2.1. Participants

We employed existing data from the PIAAC 2012 conducted by the Organisation for Economic Co-operation and Development (OECD). In total 81,744 participants aged 16 to 65 from 17 countries participated in the PIAAC test (OECD 2013). The participants were randomly assigned to two of the three cognitive modules, each of which comprised either literacy, numeracy, or problem-solving in TRE (PSTRE) tasks (OECD 2013). We analyzed 10,806 participants who responded to two PSTRE modules from 14 of the 17 countries, as data from three countries (i.e., France, Italy, and Spain) were not available. We cleaned the invalid data as some participants merely pressed the next button without responding to the questions. Participants with outliers in terms of three variables (i.e., the timing of the first action, the total number of interactions, and the duration of the entire problem-solving process) were also excluded. Outliers were identified by examining whether values lay outside of three standard deviations of the average value. Eventually, *N* = 7516 participants with an average age of 36.29 years (*SD* = 13.62) were included in the analysis, of which 47.90% were male. The demographic information of participants included in the study was presented in Table 1 by country.

### 2.2. Instruments

**Figure 1:**
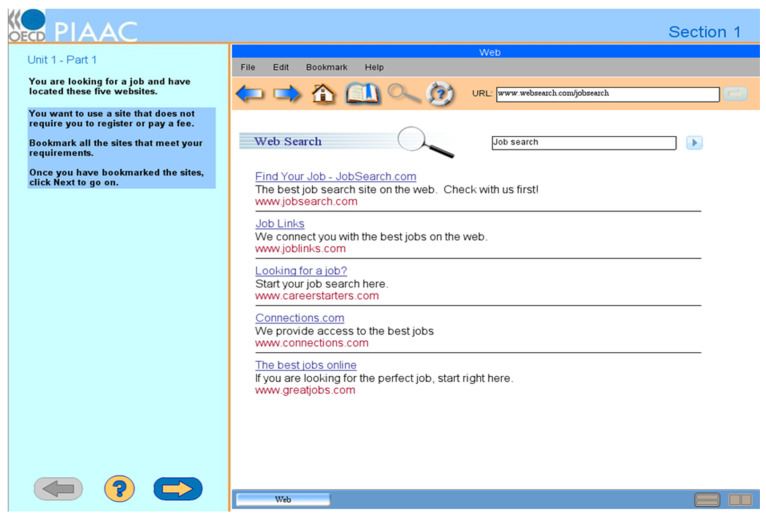
This is an exemplary problem-solving item in TRE. From Job Search Part I, by (OECD n.d.) (https://piaac-logdata.tba-hosting.de/public/problemsolving/JobSearchPart1/pages/jsp1-home.html) (accessed on 11 August 2021).

The PSTRE domain aims to measure “abilities to solve problems for personal, work and civic purposes by setting up appropriate goals and plans and accessing and making use of information through computers and computer networks” (OECD 2013, p. 56). Accordingly, 14 computerized tasks were developed to mimic real-life problems that adults are likely to encounter while using computers and Internet-based technologies (OECD 2019). OECD (2012, p. 48) defined three core dimensions when developing the 14 tasks. The first dimension is problem circumstances that trigger a person’s curiosity about problem solving and determine actions required to be taken to solve problems. The second is technologies through which problem solving is conducted, such as computer devices, applications, and functionalities. The third dimension is cognitive processes underlying problem solving (e.g., goal setting and reasoning). These three dimensions played an intertwined role in distinguishing participants’ proficiency levels in PSTRE. For example, the “Job Search” task (see Figure 1) creates a scenario in which participants assume that they are taking the role of job seekers. Participants click on links or forward/back icons and then bookmark as many web pages as possible. If participants solve this task, it is assumed that they can identify problem goals and operate technology applications. Three proficiency levels of PSTRE in total were distinguished in the PIAAC 2012 and 14 tasks were distributed over three difficulty levels (OECD 2019). More challenging tasks have higher difficulty levels: three, seven, and four tasks were at difficulty levels 1, 2, and 3 correspondingly. All participants finished each PSTRE module within 30 min. The order of tasks within each module and that of the modules were always the same. Participants were not allowed to return to a former task after finishing it.

### 2.3. Scoring

#### 2.3.1. Task Rubric and Scoring

According to the PIAAC technical report (OECD 2016), it is based on predefined scoring rubrics to grade participants’ responses. As shown in Table 2, task scores are of mixed formats: eight tasks were dichotomously scored (i.e., correct, incorrect), and six tasks were polytomously scored (i.e., full, partial, no credit).

#### 2.3.2. Behavioral Indicators Scoring

To address our research questions, planning duration and interaction frequency were extracted as behavioral indicators from log file data for the 14 PSTRE tasks in the PIAAC 2012. We used the time between participants’ view of the task and their first interaction as a measure of planning duration for one task. Thus, we had 14 measures of planning duration for each participant. Table 3 shows the descriptive statistics of these measures ranging from 0 to 16.28 min. The mean planning duration ranges from 0.26 min (*SD* = 0.19) to 0.82 min (*SD* = 0.49) for the 14 tasks. Planning durations of all tasks are almost normally distributed based on skewness values ranging from 0.72 to 1.90 (George and Mallery 2010) except for the eighth task with a skewness value of 11.72. The extremely long planning duration (16.28 min) may explain its highly skewed distribution.

For the behavioral indicator of interaction frequency, we calculated the ratio of the total number of human–computer interactions to the overall timing of interactions. The ratio was used because it normalizes the number of interactions for the timing. In addition, the ratio corresponds to core features that can distinguish different problem-solving styles effectively. The Appendix A displays a sample log data file that records sequences of actions undertaken by one participant of the PIAAC 2012. The log data file contains four variables associated with the problem-solving process in TRE. The “Item Name” variable indicates which task it is. Both the “Event Name” and “Event Type” variables explain behavioral events, which may be either system-generated (e.g., START, NEXT_ITEM, and END) or respondent-generated (e.g., CONFIRMATION_OPENED, MAIL_VIEWED, FOLDER_VIEWED). The “Timestamp” variable is the behavioral event time for the task given in milliseconds since the beginning of the assessment. We can infer that the respondent spent 0.24 min planning solutions and 2.94 min interacting with the task. Note that the overall timing of interactions is the duration from the first event to the end of the task (i.e., 2.94 min) instead of the overall timing of solving the problem (i.e., 3.18 min). Given that the total number of interactions was 45, the interaction frequency for this participant on the first task was 15.31 times/min. Similarly, we had 14 measures of interaction frequency for each respondent. As presented in Table 4, the mean interaction frequency ranged from 5.56 times/minute (*SD* = 3.30) to 18.53 times/minute (*SD* = 9.43). The skewness values show that the interaction frequencies for all tasks are normally distributed (George and Mallery 2010). It should be noted that the values of planning duration and interaction frequency did not share a common measurement scale. We thus rescale both variables using their ranges to compensate for the effect that different variations of planning duration and interaction frequency had on the following analysis (i.e., *k*-means clustering, (Henry et al. 2005)) results.

### 2.4. Data Analysis

We first conducted *k*-means clustering with planning durations and interaction frequencies to categorize participants into different problem-solving styles groups. *k*-means clustering is one of the simplest learning algorithms for sample clustering. Using *k*-means clustering, one must first fix prior k-centroids and then assign each observation to the cluster associated with its nearest centroid (Jyoti and Singh 2011). We chose this algorithm for two reasons: first, the results of *k*-means clustering analysis are feasible to interpret because clusters can be distinguished by examining what respondents in each cluster have in common regarding their behavioral patterns; second, *k*-means clustering is efficient in terms of running-time even with a large number of participants and variables, which renders applications in large-scale assessments likely (He et al. 2019). One challenge to *k*-means clustering is to figure out the number of clusters in advance. We applied the average silhouette method to determine the optimal number of clusters (e.g., Kaufman and Rousseeuw 1990). Specifically, the average silhouette method calibrated the silhouette width to measure the difference between within-cluster distances and between-cluster distances. Kodinariya and Makwana (2013) compared six methods to automatically generate the optimal number of clusters, among which the average silhouette method had been recommended because it best improved the validation of the analysis results (Kaufman and Rousseeuw 1990). We thus employed the largest average silhouette width over different *k*s to identify the best number of clusters. Additionally, we used the NbClust method (Charrad et al. 2014) to validate the result from the average silhouette method. The NbClust method aims to gather all available indices of a data set (i.e., 30 indices), as presented by Charrad et al. (2014), to generate the optimal number of clusters. Using different combinations of cluster numbers, distance measures, and clustering methods, the NbClust method outputs a consensus on the best number of clusters for the data set.

*k*-means clustering employing the average silhouette method was first implemented using the package *factoextra* (Kassambara and Mundt 2020) in R (R Core Team 2022). We then used the *NbClust* package to validate the number of clusters from the average silhouette method. Next, the average scores on planning duration (i.e., 14 indicators) and interaction frequency (i.e., 14 indicators) were compared across clusters by one-way analysis of variance (ANOVA) separately to verify *Acting*/*Reflecting* styles in TRE, which was conducted using the *dplyr* package (Wickham et al. 2021) in R (R Core Team 2022).

**Figure 2:**
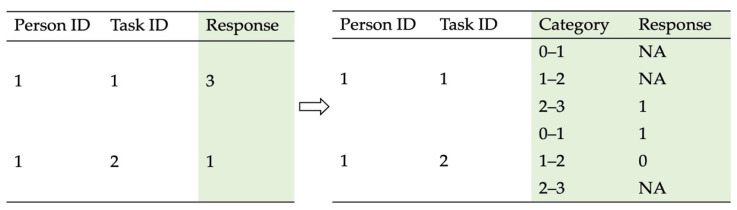
Examples of how polytomous and dichotomous responses are defined as pseudo-dichotomous responses.

EIRM was finally applied to understand the association between participants’ problem-solving styles derived from the *k*-means clustering analysis and their performance on PSTRE and how consistent the association was across multiple item difficulty levels. Unlike traditional item response theory models that solely focus on the difficulty levels of individual items, EIRM allows task-level and person-level features as well as their interactions to be incorporated into measurement models in order to explain the variation in task difficulties (De Boeck and Wilson 2004). This study employed a series of EIRM analyses, in which individuals’ problem-solving styles identified by the *k*-means clustering were the person-level predictors, and task difficulty levels were the task-level predictors of participants’ likelihood of completing the tasks correctly. We compared model fit indices and model variable coefficients to identify the most desired problem-solving style in TRE for participants. All EIRM analyses were implemented using the package *eirm* (Bulut 2021; Bulut et al. 2021) within the R computing environment (R Core Team 2022). Tasks with varying numbers of response categories were handled by the *polyreformat* function of the *eirm* package. Specifically, the *polyreformat* function transforms dichotomous and polytomous responses into a series of dummy-coded responses (Bulut et al. 2021). Figure 2 demonstrates how polytomous (i.e., task 1) and dichotomous response categories (i.e., task 2) are dichotomized in the new data set. For example, if a respondent had the response category of 3 for task 1, then the dummy-coded responses for this polytomous response would be 1 for 2–3 and missing (i.e., NA) for 0–1 and 1–2. If the respondent had the response category of 1 for task 2, then the dummy-coded responses for this dichotomous response would be 1 for 0–1, 0 for 1–2, and missing (i.e., NA) for 2–3. This series of dummy-coded responses can be performed with EIRM analyses together.

## 3. Results

### 3.1. Are Acting and Reflecting Styles Applicable to Describe Problem-Solving Styles in TRE by Examining Planning Duration and Interaction Frequency?

**Figure 3:**
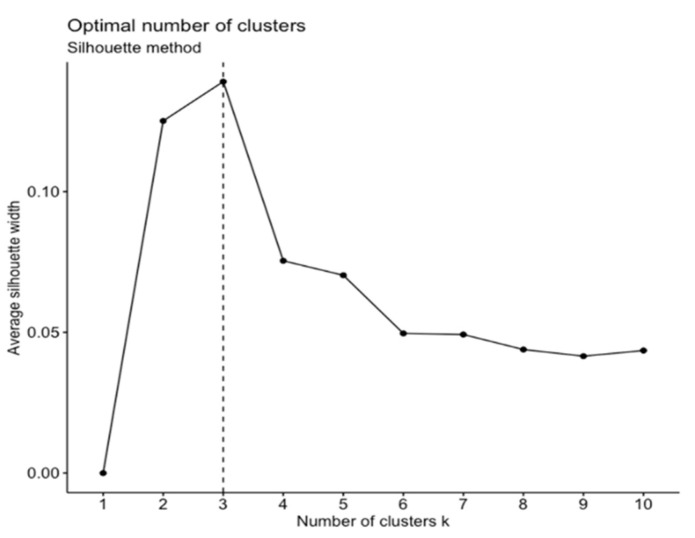
The optimal number of clusters by the average silhouette method for the two behavioral indicators.

We first used the average silhouette method to find the optimal number of clusters for the rescaled data. Figure 3 depicts the relationship between the average silhouette width and the cluster number ranging from one to ten. The three-cluster solution had the greatest silhouette width, suggesting that participants should be clustered into three groups based on their planning duration and interaction frequency on the 14 PSTRE tasks.

**Figure 4:**
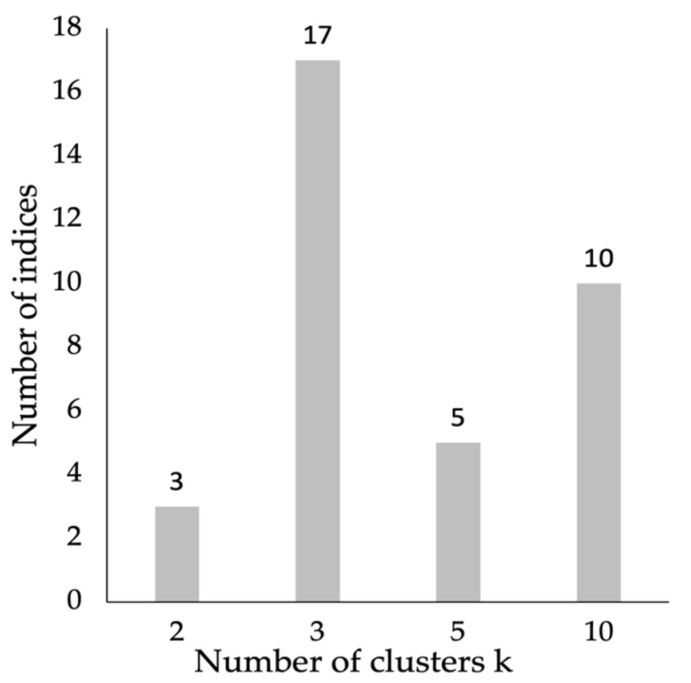
The optimal number of clusters suggested by the majority rule of the *NbClust* package for the two behavioral indicators.

To validate the three-cluster solution, we employed the NbClust method to generate a consensus on the optimal number of clusters for the data set. Figure 4 showed that the three-cluster solution was the one that was supported by most indices (i.e., 17).

**Figure 5:**
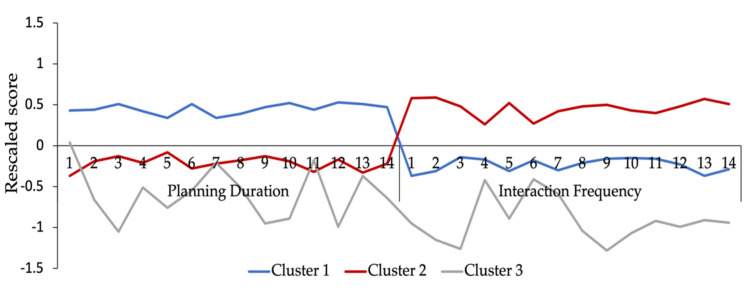
Behavioral profiles of the three clusters on the two behavioral indicators.

To understand behavioral profiles for the three clusters, rescaled scores on planning duration and interaction frequency across the three clusters were shown in Figure 5. The larger the values were, the longer the planning duration or the higher interaction frequency that participants initiated. The mean rescaled scores on planning duration are 0.45 (*SD* = 0.06), −0.22 (*SD* = 0.08), and −0.59 (*SD* = 0.33) and the mean rescaled scores on interaction frequency are −0.24 (*SD* = 0.08), 0.46 (*SD* = 0.10), and −0.92 (*SD* = 0.27). Cluster 1 suggests the highest rescaled score on planning duration, but a lower rescaled score on interaction frequency, indicating that members of this cluster spent a particularly long time in action planning and did not devote much to the interaction with technology-based problems. In contrast, cluster 2 indicates the highest rescaled score on interaction frequency, but a lower rescaled score on planning duration, revealing that participants spent less time on setting up plans while actively interacting with TRE. Unlike clusters 1 and 2, cluster 3 suggests the lowest rescaled scores of both planning duration and interaction frequency. That is, respondents in cluster 3 barely spent time making plans before the operations that followed, and they were less frequently interacting with problem-solving tasks to solve problems.

As shown in Table 5, of the participants, 2993 (39.82%), 3522 (46.86%), and 1001 (13.32%) were in clusters 1, 2, and 3, respectively. The mean values of planning duration and interaction frequency of the three clusters were also presented in Table 5. That is, solvers’ planning duration for each PSTRE task was found to be 41.06 s for cluster 1 and decreased progressively to 26.70 and 19.50 s for clusters 2 and 3. The magnitude of interaction frequency for cluster 3 (5.14 times/min) was found to be lowest in comparison with cluster 1 (10.04 times/min) and cluster 2 (14.84 times/min). Two one-way ANOVAs were performed with solvers’ clusters as the independent variable. Results indicated that differences in both behavioral indicators were significant across the three clusters, *F*(2, 7513) = 4401, *p* < .001, eta-squared = 0.540 and *F*(2, 7513) = 7609, *p* < .001, eta-squared = 0.670. Post hoc comparisons using the Tukey HSD method indicated that the planning duration of cluster 1 was the longest and the interaction frequency of cluster 2 was the highest among the three clusters. Thus, the behavioral patterns of clusters 1 and 2 were consistent with how individuals with *Reflecting* and *Acting* styles are expected to perform in TRE. We defined the problem-solving style of Cluster 3 as *Shirking* given its shortest planning duration and lowest interaction frequency.

### 3.2. How Problem-Solving Styles Are Associated with Participants’ Performance in PSTRE and How Does Task Difficulty Level Moderate Their Relationship?

To understand how task difficulty levels moderate the relationship between identified problem-solving styles in TRE and individual problem-solving performance, we conducted a series of EIRM analyses.

Model 0 represents the baseline model in which the only predictor was task difficulty levels at the task level. Difficulty scores of the 14 tasks reported by OECD (2019) were presented in Appendix B. We noted that tasks at the same difficulty level have close difficulty scores, while tasks at different difficulty levels differ greatly in their difficulty scores. The average difficulty score of tasks at difficulty level 2 (i.e., 311.7) lay outside of three standard deviations of the average difficulty score of tasks at difficulty level 1 (i.e., 274.0). It is the same when comparing tasks at difficulty level 3 with those at difficulty level 2. These pieces of information can corroborate Model 0. Model 1, as compared to Model 0, includes problem-solving styles as an additional predictor at the personal level. Lastly, Model 2 further incorporated the interaction between task difficulty and problem-solving style. The estimated parameters of Models 0, 1, and 2 are shown in Table 6. The baseline model (Model 0) shows that the estimated coefficients for task difficulty levels (TDL) are aligned with the PIAAC’s categorization of task difficulty, where level 1 represents the easiest tasks (*b* = −0.53) and level 3 indicates the hardest tasks (*b* = 1.92). The next model, Model 1, compared the three clusters with different problem-solving styles: when compared with the *Reflecting* group (reference category), participants with the problem-solving style of *Shirking* were less likely to solve PSTRE tasks correctly (*OR* = 0.17; 83% less likely), whereas participants with the problem-solving style of *Acting* had a much higher chance of conducting the PSTRE tasks correctly (*OR* = 1.58; 58% more likely). The final model, Model 2, included two-way interactions between problem-solving styles and task difficulty levels. The interaction effects were statistically significant, but very small in magnitude, suggesting that task difficulty did not strongly moderate the relationship between problem-solving styles and participants’ likelihood of solving TRE-related tasks. To directly compare the *Shirking* and the *Acting* group, we built another model (i.e., Model 1_Acting) including problem-solving styles as a predictor at the personal level and task difficulty levels as a predictor at the task level. Model 1_Acting is different from the current Model 1 because the control group in Model 1_Acting is *Acting* rather than *Reflecting*. We thus obtained the contrast between the *Shirking* and the *Acting* style: participants with the problem-solving style of *Acting* were more likely to solve PSTRE tasks correctly in comparison with those with the *Shirking* style (*z* = 63.70, *p* < 0.001). Given that Model 1_Acting was built to compare the *Shirking* and the *Acting* style, we did not include the results of Model 1_Acting in Table 6 to keep EIRM analysis results in their current flow.

Table 7 shows a summary of the three explanatory item response models. The models were compared using the relative model fit indices of the Akaike Information Criterion (AIC; Akaike 1987) and Bayesian Information Criterion (BIC; Schwarz 1978). The model fit indices indicated that Model 2 had the best fit with the smallest AIC and BIC values. Since Models 0 and 1 were nested within each other, a direct comparison between the models was made using the likelihood ratio (LR) test. Given the significant improvement in model fit (D = 5827, *p* < .001) and a large reduction in residual variance (0.24) from Model 0 to Model 1, we could statistically infer participants’ problem-solving styles explained their PSTRE performance. Similarly, the LR test between Model 1 and Model 2 was also significant (D = 59.4; *p* < .001). However, residual variance did not change from Model 1 to Model 2, indicating that the interaction effects included in Model 2 did not contribute to the model significantly. These results suggest that the advantageous effect of the *Acting* style and the disadvantageous impact of the *Shirking* style on PSTRE performance were consistent regardless of how difficult PSTRE tasks were.

## 4. Discussion

This study aimed to develop a novel understanding of what types of problem-solving styles humans exhibit in TRE using log file data and how the styles identified are associated with humans’ performance in TRE. The results disclosed three types of problem-solving styles in TRE: *Acting*, *Reflecting*, and *Shirking*. We also found the superiority of the *Acting* style as well as the inferiority of the *Shirking* style for technology-based problem solving, irrespective of problem difficulties.

Our results contribute to the current literature in several ways. First, the presence of the *Acting* and *Reflecting* styles provides new evidence to support that learning modes are associated with humans’ dispositions to solve problems in TRE. We found that some participants prefer to be involved in operations and explorations with problem environments, while others prefer to observe rather than act in technology-based problem scenarios. These inclinations are aligned with participants’ preference for action (i.e., *Acting*) or reflection (i.e., *Reflecting*) when they process information (Kolb and Kolb 2009; Richmond and Cummings 2005). This is likely because information processing is commonly involved in the problem-solving process (Reed and Vallacher 2020; van Gog et al. 2020). As Simon (1978) argued, the problem-solving process can be understood from an information-processing perspective. Thus, learning modes could serve as a stepping stone to understanding and profiling participants’ dispositions towards problem solving in TRE.

Second, the *Shirking* style expands our knowledge of humans’ dispositions towards problem solving in TRE. The participants adhering to the style of *Shirking* displayed a behavioral preference of scarcely pondering at the beginning of problem solving and barely exploring a problem scenario during the problem-solving process. Unlike the *Acting* and *Reflecting* styles, the *Shirking* style is a newly emergent style that describes participants’ avoidance of planning and actions in problem solving in TRE (D’Zurilla and Chang 1995; Shoss et al. 2016). To construct a deeper understanding of the *Shirking* style, we examined the average response time of the three style groups and found that the *Shirking* style group spent less time (1.19 min) than those with the *Acting* style (2.95 min) or *Reflecting* style (2.51 min). However, the average response time was far longer than five seconds, which was used as a constant threshold for the minimum amount of time needed to validly respond to a task (e.g., Goldhammer et al. 2016; Wise and Kong 2005). In this respect, the *Shirking* style is different from disengaged test-taking behavior, though being disengaged is common in low-stakes assessments, such as the PIAAC 2012 (Goldhammer et al. 2016; Ulitzsch et al. 2021). Since various factors (e.g., cognition and personality) may impact how people respond to technology-based problems (Feist and Barron 2003), future studies should collect more data to explore what factors are associated with the presence of the three problem-solving styles in TRE.

Third, by comparing the three problem-solving styles, we are able to better understand the role of early planning and explorations in problem solving in TRE. Participants with an *Acting* style outperformed the other participants in problem solving in TRE, which confirms the assertion that actively initiating action may be a requisite for solving problems (Kolb and Fry 1975). When participants explore problem scenarios, including intuitive trial and error and stable routines within simulated computer platforms, they would gain the necessary information for problem solving, and thus enhance their chances of finding correct solutions (Liu et al. 2011). Eichmann et al. (2019) suspected that challenging tasks may require tryouts before meaningful planning. In this study, we found that participants with the *Reflecting* style were able to solve problems at difficulty levels 1 and 2, while those with the *Acting* style were able to solve more challenging problems, at all difficulty levels 1–3. This finding indicates that persistent trials play a more critical role than early planning in conducting difficult tasks. Further, in this study, the *Acting* style group differed from the *Reflecting* style group in the rescaled interaction frequency (0.73 higher) and planning duration (0.79 lower), indicating that high interaction frequency might make up for a short planning duration when participants solved technology-related problems, not vice versa.

We also noted some limitations of the present study. First, we did not explore participants excluded from this study due to outliers. Removed participants might take time to think or plan but finally skip an item. Furthermore, excluded participants might give up or abandon any explorations at the beginning of an item. These patterns barely reveal individuals’ problem-solving styles in TRE, which have been defined as dispositions regarding how they are inclined to interact with surrounding technology environments in this study. However, their relationship to motivation when participants performed the low-stakes PSTRE assessment could be investigated in future studies. Second, it is actually not known how the time between participants’ view of a task and their first interaction is actually used for planning. Eichmann et al. (2019) used the duration of the longest interval between two successive interactions to define planning. However, Albert and Steinberg (2011) argued that individuals complete their initial planning phase before taking their first interaction with a task. Thus, additional work is needed to further explore the mapping of implicit planning processes. Third, we only abstracted planning duration and interaction frequency from log files corresponding to the *Acting* and *Reflecting* styles. Other learning styles described in ELT, such as Feeling and Thinking, were not included. Thus, this study partially confirms the applicability of ELT in describing problem-solving styles in TRE. Future research may include additionally detailed behavioral and/or cognitive information so that other styles and their potential link with PSTRE performance can be figured out. Fourth, this study only examined interaction effects between problem-solving styles and task difficulty levels on participants’ performance, so future studies could include other critical cognitive factors, such as respondents’ literacy and numeracy ability. As suggested by Xiao et al. (2019), cognitive factors may interact with participants’ problem-solving styles and collectively act on individuals’ problem-solving performance in TRE. Future studies could continue to explore potential interactions using the present research framework.

To summarize, this study provides critical evidence for the dominant role of active explorations in solving technology-based problems. The participants were adults so the knowledge generated in this study would help improve adult education programs, as well as computer-assisted problem-solving practice systems. As Ibieta et al. (2019) indicated, providing more detailed and specific cues (e.g., if you need to view emails, please click on this button) to facilitate participants’ explorations and operations may be an effective approach in improving adults’ problem-solving proficiency in TRE.

## Figures and Tables

**Table 1:** Demographic Information of Participants in the Present Study.

Country	*N*	Gender		Age	
		Male	Female	Average	*SD*
Austria	414	227	187	NA ^1^	NA ^1^
Belgium	503	255	248	37.29	13.74
Denmark	684	316	368	42.31	14.44
Estonia	628	283	345	35.73	13.21
Finland	501	264	237	37.44	13.22
Germany	420	206	214	NA ^1^	NA ^1^
Ireland	492	236	256	36.94	11.77
Republic of Korea	465	226	239	33.71	11.84
Netherlands	521	242	279	39.11	14.49
Norway	496	253	243	37.96	13.54
Poland	711	352	359	26.25	9.90
Slovakia	383	197	186	33.57	12.99
United Kingdom	869	338	531	38.51	12.91
United States	429	205	224	NA ^1^	NA ^1^

^1^ NA indicates there is no available information.

**Table 2:** Scoring Types and Scores of the 14 Tasks.

Task	Type	Scores
1	P	0, 1, 2, 3
2	D	0, 1
3	P	0, 1, 2, 3
4	D	0, 1
5	P	0, 1, 2, 3
6	D	0, 1
7	D	0, 1
8	D	0, 1
9	P	0, 1, 2, 3
10	D	0, 1
11	D	0, 1
12	P	0, 1, 2
13	D	0, 1
14	P	0, 1, 2, 3

Note: D indicates the task is dichotomously scored. P denotes the task is polytomously scored.

**Table 3:** Descriptive Statistics of Planning Duration Indicator for 14 Tasks.

Task	Planning Duration (minutes)				
	M	*SD*	Min	Max	Skewness
1	0.56	0.34	0.00	2.51	1.52
2	0.48	0.28	0.00	1.68	0.72
3	0.38	0.25	0.00	1.75	1.10
4	0.72	0.49	0.00	5.47	1.70
5	0.57	0.56	0.00	3.86	1.90
6	0.82	0.49	0.00	2.96	0.95
7	0.33	0.25	0.00	1.39	1.03
8	0.52	0.38	0.00	16.28	11.72
9	0.26	0.19	0.00	1.16	1.13
10	0.43	0.28	0.00	1.65	0.90
11	0.79	0.62	0.00	3.58	1.58
12	0.54	0.37	0.00	2.03	0.78
13	0.55	0.29	0.00	1.90	0.89
14	0.39	0.24	0.00	1.42	0.80

**Table 4:** Descriptive Statistics of Interaction Frequency Indicator for the 14 Tasks.

Task	Interaction Frequency (times/minute)				
	M	*SD*	Min	Max	Skewness
1	18.53	9.43	0.00	103.65	0.19
2	16.46	8.03	0.00	42.09	−0.30
3	11.25	6.42	0.00	34.55	0.25
4	8.27	5.74	0.00	28.85	0.99
5	10.87	9.45	0.00	86.26	1.29
6	5.56	3.30	0.00	20.19	1.30
7	6.36	3.97	0.00	20.67	0.60
8	11.48	4.96	0.00	27.38	−0.28
9	17.11	10.59	0.00	58.27	0.05
10	10.96	6.67	0.00	33.27	0.47
11	18.25	10.15	0.00	50.40	0.31
12	6.75	5.12	0.00	25.43	0.72
13	8.21	3.56	0.00	19.18	−0.03
14	12.85	7.08	0.00	46.10	0.45

**Table 5:** Summary of Two Behavioral Indicators of Each PSTRE Task for Three Clusters.

Cluster ID	N	Planning Duration (s)	Interaction Frequency (times/min)
1	2993	41.06	10.04
2	3522	26.70	14.84
3	1001	19.50	5.14

**Table 6:** A summary of EIRM results for Model 0, Model 1, and Model 2.

	Model 0				Model 1				Model 2			
	*b*	*SE*	*Z*	*OR*	*b*	*SE*	*Z*	*OR*	*b*	*SE*	*Z*	*OR*
TDL 1	−0.53	0.02	28.06	0.59	−0.59	0.02	29.12	0.55	−0.57	0.02	23.32	0.57
TDL 2	0.33	0.01	−24.25	1.39	0.34	0.01	−22.68	1.41	0.34	0.02	−21.26	1.41
TDL 3	1.92	0.02	−87.94	6.82	1.94	0.02	−86.20	6.96	1.92	0.03	−71.49	6.82
*Shirking*					−1.75	0.03	−55.33	0.17	−1.93	0.05	−37.74	0.15
*Acting*					0.46	0.02	29.42	1.58	0.56	0.03	17.69	1.75
TDL 2*Shirking									−0.34	0.06	5.43	0.71
TDL 3*Shirking									−0.02	0.11	0.14	0.98
TDL 2*Acting									0.12	0.04	−3.32	1.13
TDL 3*Acting									0.14	0.04	−3.27	1.15

Note: TDL = task difficulty level; TDL 2 or 3 indicates tasks locating difficulty level 2 or 3; *Shirking* and *Acting* were compared to the style of *Reflecting*. OR = Odds-ratio. All the estimated coefficients except for TDL 3*Shirking were statistically significant at α = .001 or α = .01.

**Table 7:** Overview of the estimated explanatory item response theory models.

Model	Predictors			AIC	BIC	Variance	LR Test		
	Task	Person	Interaction				*df*	D	Comparison
Model 0	TDL			161,860	161,959	0.42			
Model 1	TDL	PSS		156,037	156,156	0.18	2	5827 ***	with Model 0
Model 2	TDL	PSS	TDL * PSS	155,986	156,144	0.18	4	59.4 ***	with Model 1

*** *p* < .001. Note: TDL = Task difficulty level; PSS = Problem-solving style; AIC = Akaike Information Criterion; BIC = Bayesian Information Criterion; D = Deviance; LR = Likelihood ratio.

## Data Availability

The data presented in this study are not publicly available because they are confidential and proprietary (i.e., owned by the OECD). Requests to access the data should be directed to the OECD.

## Appendix A

**Table A1:** An Exemplary Log File Data Including Events and Timestamps.

Item Name	Event Name	Event Type	Timestamp
U23x000S	taoPIAAC	START	0
U23x000S	taoPIAAC	NEXT_INQUIRY	14,449
U23x000S	taoPIAAC	NEXT_BUTTON	14,449
U23x000S	stimulus	CONFIRMATION_OPENED	14,452
U23x000S	taoPIAAC	BUTTON	14,454
U23x000S	taoPIAAC	DOACTION	14,454
U23x000S	stimulus	BUTTON	24,235
U23x000S	stimulus	CONFIRMATION_CLOSED	24,236
U23x000S	stimulus	DOACTION	24,236
U23x000S	stimulus	MAIL_VIEWED	44,710
U23x000S	stimulus	MAIL_VIEWED	75,883
U23x000S	stimulus	MAIL_VIEWED	82,687
U23x000S	stimulus	MAIL_VIEWED	90,234
U23x000S	stimulus	MAIL_VIEWED	95,535
U23x000S	stimulus	MAIL_VIEWED	102,879
U23x000S	stimulus	MAIL_VIEWED	117,178
U23x000S	stimulus	MAIL_VIEWED	125,317
U23x000S	stimulus	MAIL_VIEWED	128,700
U23x000S	stimulus	FOLDER_VIEWED	141,563
U23x000S	stimulus	MAIL_DRAG	149,706
U23x000S	stimulus	MAIL_VIEWED	151,488
U23x000S	stimulus	TOOLBAR	165,881
U23x000S	stimulus	ENVIRONMENT	165,883
U23x000S	stimulus	DOACTION	165,883
U23x000S	stimulus	DOACTION	165,884
U23x000S	stimulus	DOACTION	165,884
U23x000S	stimulus	DOACTION	165,885
U23x000S	stimulus	TOOLBAR	167,934
U23x000S	stimulus	ENVIRONMENT	167,936
U23x000S	stimulus	DOACTION	167,936
U23x000S	stimulus	DOACTION	167,941
U23x000S	stimulus	DOACTION	167,942
U23x000S	stimulus	DOACTION	167,943
U23x000S	stimulus	TOOLBAR	171,676
U23x000S	stimulus	ENVIRONMENT	171,677
U23x000S	stimulus	DOACTION	171,677
U23x000S	stimulus	DOACTION	171,678
U23x000S	stimulus	DOACTION	171,679
U23x000S	stimulus	DOACTION	171,679
U23x000S	stimulus	TOOLBAR	173,631
U23x000S	stimulus	ENVIRONMENT	173,633
U23x000S	stimulus	DOACTION	173,633
U23x000S	stimulus	DOACTION	173,633
U23x000S	stimulus	DOACTION	173,634
U23x000S	stimulus	DOACTION	173,634
U23x000S	stimulus	TEXTLINK	182,570
U23x000S	stimulus	HISTORY_ADD	182,727
U23x000S	taoPIAAC	NEXT_INQUIRY	188,529
U23x000S	taoPIAAC	NEXT_BUTTON	188,529
U23x000S	stimulus	CONFIRMATION_OPENED	188,532
U23x000S	taoPIAAC	BUTTON	188,538
U23x000S	taoPIAAC	DOACTION	188,538
U23x000S	stimulus	BUTTON	190,901
U23x000S	stimulus	CONFIRMATION_CLOSED	190,902
U23x000S	taoPIAAC	NEXT_ITEM	190,904
U23x000S	taoPIAAC	END	190,905

## Appendix B

**Table A2:** Difficulty Scores and Difficulty Levels of the 14 Tasks (OECD 2016).

Task	Difficulty Score	Difficulty Level	Difficulty Range	Average (*SD*)
1	286	1	268 to 286	274.0 (10.39)
10	286			
11	268			
2	299	2	296 to 325	311.7 (11.57)
4	316			
7	325			
8	305			
12	296			
13	320			
14	321			
3	346	3	342 to 374	354.2 (14.24)
5	374			
6	342			
9	355			
